# Beyond SWS1 Cones: Canonical and Noncanonical Routes of Ultraviolet Sensitivity in the Avian Retina

**DOI:** 10.3390/birds7030050

**Published:** 2026-08-21

**Authors:** Lidia Zueva, Vassiliy Tsytsarev, Janaina Alves, Mikhail Inyushin

**Affiliations:** 1 Department of Physiology, Universidad Central del Caribe, Bayamon, PR 00960, USA; 2 Laboratory of Applied Physics, Johns Hopkins University, Baltimore, 11100 Johns Hopkins Road, Laurel, MD 20723, USA; 3 Department of Microbiology and Immunology, Universidad Central del Caribe, Bayamon, PR 00960, USA

**Keywords:** avian retina, ultraviolet vision, short-wavelength-sensitive type 1 cone, double cones, oil droplets, fluorescence, β-band, rhodopsin, opsin 5, Müller glia

## Abstract

Ultraviolet sensitivity in birds is generally attributed to short-wavelength-sensitive type 1 single cones, but ultraviolet radiation may influence the retina through several additional pathways. We reviewed molecular, optical, physiological, and behavioral evidence for canonical and putative noncanonical ultraviolet detection in the avian retina. Under photopic conditions, short-wavelength-sensitive type 1 cones provide an established chromatic pathway, whereas secondary ultraviolet absorption bands of other cone pigments may contribute at high irradiance. Under scotopic conditions, particularly in rod-dominated owls lacking functional ultraviolet- or violet-sensitive type 1 cones, the secondary ultraviolet absorption band of rhodopsin provides a plausible achromatic pathway. Ultraviolet-sensitive opsin 5 in inner-retinal neurons and Müller glia may mediate local regulatory or non-image-forming responses. We also examine fluorescence of oil droplets in double cones as a possible route into the luminance system. Previous reports described ultraviolet-induced visible fluorescence in chicken retinal oil droplets and fluorescence of chicken double-cone droplets under blue excitation, although physiological activation of the adjacent visual pigment by droplet fluorescence has not been established. These mechanisms are not mutually exclusive, and their relative contributions probably depend on irradiance, adaptation state, ocular-media transmission, receptor abundance, and behavioral context. Receptor-specific physiological and behavioral experiments are required to distinguish between them.

## Simple Summary

Birds can detect ultraviolet light, which is invisible to humans, but they may use different retinal mechanisms under different lighting conditions. This review compares ultraviolet detection by color-sensitive cones in daylight, highly sensitive rods at night, light-sensitive molecules in retinal nerve and supporting cells, and fluorescent oil droplets in double cones. Evidence suggests that these pathways may complement one another rather than provide a single universal mechanism. Understanding them may improve knowledge of bird vision, behavior, environmental adaptation, and responses to artificial lighting.

## 1. Introduction

Birds possess one of the most complex vertebrate retinas. A typical avian retina contains rods, four classes of single cones, and double cones composed of closely associated principal and accessory members [1-3]. These receptors support vision across a wide range of illumination conditions and allow wavelength, luminance, spatial, and temporal information to be processed through partly specialized pathways.

Rods provide high-sensitivity vision under dim, scotopic conditions, whereas cones provide faster responses, higher temporal resolution, greater spatial resolution, and spectral discrimination under brighter, photopic conditions [4,5]. The four avian single-cone classes express the visual pigments SWS1, SWS2, RH2, and LWS and constitute the receptor basis for tetrachromatic color vision. Double cones generally express LWS-family pigments and have been associated primarily with achromatic luminance and motion-related processing [1,3,6,7].

Ultraviolet sensitivity is usually attributed to the SWS1 single cone. However, behavioral detection of ultraviolet radiation does not necessarily identify the photoreceptor or retinal pathway responsible. Ultraviolet photons may also activate secondary absorption bands of visual pigments, including rhodopsin, or be detected by nonvisual opsins expressed in retinal neurons and glial cells. In addition, fluorescence within cone oil droplets could convert ultraviolet photons into visible photons that might be captured by visual pigments whose principal sensitivity lies outside the ultraviolet range.

These mechanisms are not mutually exclusive. Their relative importance may vary with illumination intensity, adaptation state, species, age, ocular-media transmission, retinal region, and behavioral task. Accordingly, the term “ultraviolet sensitivity” may encompass fundamentally different processes, including chromatic ultraviolet vision, achromatic ultraviolet-enhanced luminance vision, rod-mediated scotopic detection, and non-image-forming retinal photoreception.

This review aims to evaluate evidence for canonical and putative noncanonical routes of ultraviolet detection in the avian retina. We compare the illumination conditions, receptor types, and potential functional outputs associated with these pathways and identify experimental approaches that may distinguish between them. Attention is given to the fluorescence of the P-type oil droplet in the double cone as a possible pathway by which ultraviolet radiation could influence the achromatic luminance system.

## 2. Canonical and Putative Noncanonical Pathways and Evidence for Ultraviolet Sensitivity in Birds

### 2.1. The Canonical SWS1-Cone Pathway

The canonical avian ultraviolet pathway is mediated by the SWS1 single cone. Depending on the amino acid sequence of its visual pigment, the SWS1 cone may be ultraviolet-sensitive, with maximal sensitivity typically between 355 and 380 nm, or violet-sensitive, with maximal sensitivity above 400 nm [2,8]. Relatively small amino-acid substitutions at important spectral-tuning sites can shift the SWS1 absorption maximum between ultraviolet-sensitive and violet-sensitive forms [8-10].

The SWS1 cone usually contains a transparent T-type oil droplet with little measurable absorption across the ultraviolet and visible spectrum. Unlike the strongly colored droplets associated with other cone classes, the T-type droplet permits short-wavelength photons to reach the SWS1 outer segment with minimal additional filtering [2,10].

Effective ultraviolet sensitivity nevertheless depends on more than the presence of a UV-sensitive opsin. Ultraviolet radiation must first pass through the cornea, lens, and vitreous. Ocular-media transmission differs substantially among birds and may also change with age. A retina containing a UV-sensitive SWS1 pigment will have limited ultraviolet sensitivity if the lens strongly absorbs the relevant wavelengths [11-13].

Signals from the SWS1 cone can be compared with signals from SWS2, RH2, and LWS single cones in post-receptoral opponent circuits. This comparison allows the visual system to distinguish changes in wavelength from changes in overall illumination. Canonical SWS1-mediated UV vision can therefore support chromatic discrimination and contribute to foraging, orientation, mate selection, offspring recognition, and detection of ultraviolet-reflecting plumage, skin, or food [2,14,15].

Most direct behavioral demonstrations of avian ultraviolet color vision have been obtained in diurnal birds under daylight or controlled photopic illumination. Under these conditions, cone-mediated vision is expected to dominate, and the SWS1 pathway provides the most parsimonious explanation when a functional UVS or VS SWS1 pigment is present.

### 2.2. The Canonical Double-Cone Pathway

Avian double cones consist of a larger principal member and a smaller accessory member that are closely apposed along much of their length. The two members possess distinct outer segments, nuclei, and synaptic structures, but they appear to form a coordinated receptor unit. In most birds studied, both members express LWS-family visual pigments [1,16,17].

The principal member contains a P-type oil droplet, which may appear pale or greenish, positioned between the ellipsoid and the outer segment. The occurrence of an oil droplet in the accessory member differs among species. Accessory-member droplets are present in chickens and pigeons but may be absent in turkeys and in some retinal regions of European robins [17,18]. Such variation suggests that the optical properties of double cones may be adapted to species-specific visual environments.

Double cones are often the most abundant cone type in the avian retina. Their relative abundance varies among species and retinal regions, but they commonly constitute approximately 40–50% of the total cone population. In the chicken retina, double cones account for approximately 40.7% of all cones [1,3], a species-specific value within this broader range. Their high abundance and, in particular, their regular retinal mosaic are consistent with a major role in spatially extensive visual processing.

Double cones have been associated primarily with achromatic luminance and motion detection rather than tetrachromatic color vision [3,7]. Additional roles in polarization sensitivity and magnetoreception have been proposed based on the oriented organization of the double-cone mosaic [19]. However, these proposed functions remain unconfirmed, and the downstream circuitry and complete functional repertoire of double cones are incompletely understood [17-19].

Because LWS visual pigments have their principal absorption maxima in the medium-to-long-wavelength region, double cones are not conventionally classified as ultraviolet receptors. Nevertheless, ultraviolet radiation could influence their output through at least two mechanisms: direct absorption by the secondary ultraviolet β-band of the LWS pigment and indirect activation following wavelength-shifting fluorescence within the P-type oil droplet.

### 2.3. Cone Organization and Oil Droplets in the Avian Retina

**Figure 1.**
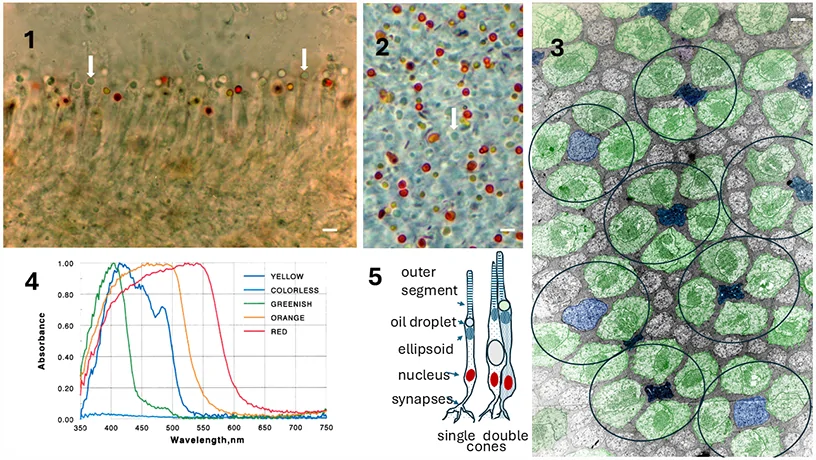
Cone photoreceptors and oil droplets in the retina of the pied flycatcher (*Ficedula hypoleuca*). (**1**) Light micrograph of a vertical retinal section showing cone photoreceptors with differently colored oil droplets in their inner segments. Arrows indicate pale-green droplets tentatively identified as P-type droplets of double-cone principal members based on their size, color, and position. (**2**) En face light-microscopic view of the photoreceptor mosaic at the level of the oil droplets. The arrow indicates a pale-green P-type droplet. (**3**) Transmission electron micrograph showing double cones highlighted in green and selected single cones containing blue droplets highlighted in blue. The colors are overlays added to identify the receptor classes and are not natural electron-microscopic colors. Multiple double cones frequently form rosette-like arrangements around single cones. Panels (**1**–**3**) are previously unpublished images obtained during the study reported in [24]. Briefly, retinal tissue was examined in vertical sections and “en face” preparations by light microscopy and in ultrathin sections by transmission electron microscopy. Oil droplets and photoreceptor classes were identified from their position, morphology, optical properties, and ultrastructural characteristics. (**4**) Representative normalized absorbance spectra of different oil-droplet types, adapted from [24]. Strongly colored droplets act as long-pass filters, whereas pale or apparently colorless droplets transmit a broad range of short wavelengths. (**5**) Schematic organization of single and double cones showing the position of the oil droplet immediately proximal to the outer segment. Bars: 10 μm (in (**1**,**2**)), 1 μm (in (**3**)).

As a representative example, we show in Figure 1 the morphology and organization of cone photoreceptors in the retina of the pied flycatcher (*Ficedula hypoleuca*).

Panel 1 shows a vertical retinal section containing differently colored oil droplets within the inner segments of cone photoreceptors. The pale-green droplets indicated by arrows were tentatively identified as the P-type droplets of double-cone principal members based on their size, color, and position. The droplets lie immediately proximal to the outer segments, so incident light normally passes through them before reaching the visual pigments [20,21]. Panel 2 shows a “face” view of the photoreceptor mosaic at the level of the oil droplets. Red, orange, yellow, blue, and pale-green droplets can be distinguished by their color and optical properties. The pale green P-type droplets associated with double cones are distributed repeatedly across the retinal surface. Panel 3 shows the photoreceptor organization by transmission electron microscopy. Double cones are highlighted in green, whereas selected single cones containing blue droplets are highlighted in blue. The principal and accessory members remain separated by their plasma membranes. Multiple double cones frequently surround individual single cones, creating rosette-like local arrangements. Similar organized double-cone mosaics have been described in other avian retinas [19]. Panel 4 presents representative absorbance spectra of different oil-droplet classes. Yellow, orange, and red droplets strongly absorb shorter wavelengths and function as long-pass spectral filters. In contrast, pale or apparently colorless droplets transmit a broad range of short wavelengths [22,23]. Absorbance measurements, however, indicate only how much light is removed from the directly transmitted beam. They do not determine whether the absorbed energy is dissipated nonradiatively or partially re-emitted as fluorescence. Panel 5 illustrates the position of the oil droplet relative to the outer segment. Because the P-type droplet is located immediately proximal to the outer segment of the double-cone principal member, visible fluorescence generated within the droplet would originate near the LWS visual pigment. This geometry could allow a fraction of the emitted photons to enter the outer segment.

### 2.4. Behavioral Evidence for Ultraviolet Perception Under Photopic Conditions

Behavioral studies provide strong evidence that birds use ultraviolet information under daylight or controlled photopic conditions. Manipulation of ultraviolet reflectance affects mate choice, foraging, offspring recognition, and other visually guided behaviors in several species [14,15,25]. In birds with functional UVS or VS SWS1 cones, the canonical SWS1 pathway offers the most direct explanation.

Rubene et al. [26] examined the critical flicker-fusion frequency of chickens using UV-free white light, UV-enriched white light, narrow-band yellow light, and narrow-band UV illumination at several intensity levels. The addition of ultraviolet wavelengths increased temporal resolution, and chickens detected the flicker produced by narrow-band UV stimulation around 400 nm. The response occurred under photopic experimental conditions and therefore demonstrates that ultraviolet illumination can influence a rapid daylight visual pathway.

This result is particularly interesting because critical flicker-fusion frequency primarily measures temporal and luminance processing rather than color discrimination. Double cones are generally associated with achromatic and motion-related processing [3,7], making them a possible contributor. However, Rubene et al. [26] did not isolate the responsible receptor. The response could have involved SWS1 cones, β-band absorption by other cone pigments, fluorescence-mediated double-cone activation, or convergence of multiple cone signals in downstream luminance circuits.

Penguins provide another potentially informative example. A UV-maximized SWS1 pigment has not been identified in penguins, although the Humboldt penguin (*Spheniscus humboldti*) possesses a functional violet-sensitive SWS1 pigment with a maximum near 403–405 nm [16,27]. Gentoo penguins (*Pygoscelis papua*) have reportedly responded behaviorally to illumination near 365 nm, and the ocular media of Gentoo and Little penguins (*Eudyptula minor*) transmit substantial near-ultraviolet radiation [13].

The penguin response could arise through the short-wavelength tail or β-band of the violet-sensitive SWS1 pigment, the β-bands of other cone pigments, or possibly fluorescence in double-cone oil droplets. The observation is consistent with near-UV detection but does not identify its receptor. Photopic testing is particularly important in penguins because it can minimize rod contributions and focus attention on cone-based mechanisms.

### 2.5. Behavioral Evidence for Ultraviolet Perception at Night

Evidence that ultraviolet-associated signals affect behavior at night has been reported in owls. Scops owl (*Otus scops*) nestlings possess strongly UV-reflective ceres, and experimental manipulation of cere reflectance altered parental food allocation during nocturnal provisioning [28]. This result indicates that a visual signal containing an ultraviolet component can influence nocturnal behavior. However, manipulating UV reflectance does not identify the photoreceptor or retinal pathway responsible for detecting the signal.

Höglund et al. [29] demonstrated that the SWS1 gene is pseudogenized in owl species examined and concluded that these birds lack functional UV/violet SWS1 cones. Nevertheless, owl ocular media transmit near-ultraviolet radiation. The authors proposed that this transmission increases rod photon catch through the secondary ultraviolet β-band of rhodopsin.

The owl retina is strongly rod-dominated. In the great horned owl (Bubo virginianus), rods, single cones, and double-cone units were reported in an approximate ratio of 30:1:2 [30]. Expressed per photoreceptor unit, rods therefore outnumber single-cone and double-cone units combined by approximately 10:1. This receptor composition, together with the large aperture of the owl eye and extensive neural summation, strongly favors rod-mediated vision at night [30,31].

Consequently, owl UV sensitivity under scotopic conditions should not be considered direct evidence of double-cone fluorescence. A rhodopsin β-band mechanism is more parsimonious. Fluorescent double cones could still contribute under mesopic or photopic conditions, but their involvement would have to be demonstrated after rod activity was experimentally separated or suppressed.

### 2.6. β-Band Absorption by Cone Visual Pigments

Visual pigments possess a dominant α-band surrounding their principal absorption maximum and a weaker β-band at shorter wavelengths. As a result, visual pigments that are maximally sensitive in the visible spectrum retain some absorption in the near-ultraviolet [32].

Under sufficiently strong ultraviolet illumination, the β-bands of VS-SWS1, SWS2, RH2, or LWS cone pigments could produce photoreceptor responses. This mechanism may be especially relevant in species that lack a UV-maximized SWS1 pigment but possess UV-transmitting ocular media, such as some penguins.

β-Band activation of cone visual pigments should be most effective under photopic or mesopic conditions, when the incident photon flux is high enough to compensate for relatively weak secondary absorption. It is therefore a plausible explanation for high-irradiance ultraviolet responses but is less likely to support vision under very dim nocturnal conditions.

The presence of β-bands in visual-pigment absorption spectra is well established, but their quantitative contribution to avian UV behavior remains incompletely demonstrated. Behavioral detection of UV alone cannot establish β-band involvement. Receptor-specific action spectra must be compared with the predicted α- and β-band absorption spectra of candidate pigments.

### 2.7. β-Band Absorption by Rod Rhodopsin

Rhodopsin also possesses a secondary absorption band in the ultraviolet region [32]. In species whose ocular media transmit near-UV wavelengths, sufficiently intense UV photons can activate rhodopsin even though its principal sensitivity maximum lies near 500 nm [29,32].

This mechanism is particularly plausible in owls because they lack functional SWS1 cones and possess highly rod-dominated retinas [29,30]. Rods are much more sensitive than cones, and rod pathways provide extensive spatial and temporal summation. A relatively weak ultraviolet absorption band could therefore become behaviorally significant under nocturnal conditions if enough ultraviolet photons reach the retina.

A rod-mediated UV response should be achromatic, strongest after dark adaptation, relatively slow, and suppressed when rods become saturated under bright photopic illumination. These predictions distinguish it from a rapid cone- or double-cone-mediated response.

Although rhodopsin β-band activation provides a plausible explanation for nocturnal UV sensitivity, to our knowledge, it has not been fully isolated by receptor-specific electrophysiology in owls. It should therefore be described as a well-supported candidate mechanism rather than a completely demonstrated behavioral pathway.

### 2.8. Retinomotor Movements and Illumination-Dependent Receptor Access

In many vertebrate retinas, changes in ambient illumination produce retinomotor, or photomechanical, movements involving photoreceptors and the retinal pigment epithelium. These changes include elongation and contraction of photoreceptor myoids and coordinated migration of melanin granules within the apical processes of the retinal pigment epithelium (RPE) [33,34].

In species exhibiting pronounced photomechanical responses, light adaptation generally causes cone myoids to contract and rod myoids to elongate, whereas dark adaptation produces the reverse response: cones elongate and rods contract [33,34]. Retinomotor responses can therefore function as an optical switching mechanism between cone-dominated photopic vision and rod-dominated scotopic vision. At the same time, RPE melanosomes redistribute within the apical processes surrounding the photoreceptor outer segments. Under bright illumination, melanosomes move into these processes, where they absorb scattered light and increase the optical isolation of adjacent photoreceptors. Under dim illumination, melanosomes aggregate or retract toward the RPE cell bodies, reducing the optical separation between neighboring receptors. This response may sacrifice spatial resolution while increasing light collection, an effect previously compared to “pixel binning” in digital cameras [35]. Thus, dark adaptation may increase sensitivity not only through rod dominance but also through changes in the optical coupling of neighboring photoreceptors. Conversely, the dispersion of melanosomes and greater optical isolation during light adaptation may favor spatially resolved cone-mediated vision. These effects could influence the contribution of canonical and noncanonical ultraviolet-sensitive pathways under different illumination conditions. This organization supports the expectation that cone-mediated noncanonical UV mechanisms, including cone β-bands and double-cone fluorescence, should be most effective under photopic or upper-mesopic conditions. In contrast, detection of the rhodopsin β-band should become relatively more important during dark adaptation. However, the magnitude and even the presence of particular retinomotor responses vary among vertebrate groups and species; therefore, their role in avian ultraviolet perception should be evaluated for each species rather than assumed from a general model.

Nevertheless, retinomotor movements do not necessarily make cones completely inactive at night. Their extent varies among species, and mesopic illumination can support simultaneous rod and cone activity. Moonlight, twilight, nest illumination, and artificial light may produce retinal conditions that differ substantially from complete scotopic darkness.

Retinomotor responses also cannot be assumed to occur equally in all birds. Ultrastructural studies of great horned (*Bubo virginianus*) and barred owls (*Strix varia*) found little evidence that their rods or cones undergo pronounced photomechanical movements [30,36]. Owls may instead maintain a retina structurally specialized for high scotopic sensitivity. Retinomotor enhancement should therefore be considered a species-dependent modifier of UV pathways rather than a universal explanation.

### 2.9. Ultraviolet-Induced Fluorescence in Cone Oil Droplets

Fluorescence provides a mechanism by which a photoreceptor could respond indirectly to wavelengths poorly absorbed by its visual pigment. A fluorophore absorbs a higher-energy short-wavelength photon and re-emits part of that energy as a lower-energy photon at a longer wavelength. If the emitted wavelength overlaps the absorption spectrum of a nearby visual pigment, fluorescence can extend the receptor’s effective spectral range.

Ohtsuka [37] used fluorescence microscopy and microspectrophotometric identification to distinguish cone classes according to the optical properties of their oil droplets. In the turtle retina, apparently colorless or pale-green oil droplets associated with red-sensitive cones initially produced bright greenish fluorescence under near-ultraviolet irradiation at approximately 330–380 nm; during continued observation, the fluorescence changed toward red [37]. The emission passed through a long-pass filter transmitting wavelengths above approximately 460 nm, demonstrating the conversion of near-ultraviolet excitation into visible emission.

The fluorescence was not a general property of all colorless droplets. Colorless droplets associated with other cone classes did not exhibit the same bright emission, indicating the presence of a specific fluorophore with a large Stokes shift in droplets associated with long-wavelength-sensitive cones [37,38].

Importantly, Ohtsuka [37] also reported observing the same near-ultraviolet-induced fluorescence in corresponding oil droplets of the chicken retina. Although the chicken observation was described only briefly and was not spectrally characterized in the same detail as the turtle experiments, it extended the reported phenomenon to birds [37]. However, the exact chicken oil-droplet class exhibiting this ultraviolet-induced fluorescence was not established in that brief report. The reported brightness of the fluorescence suggests that the droplets produced substantial visible emission under the experimental conditions and may have contained a relatively efficient fluorophore. However, fluorescence quantum yield was not measured, and the efficiency of ultraviolet-to-visible photon conversion therefore remains unknown.

Kram et al. [3] independently used oil-droplet fluorescence to identify chicken cone classes. Under ultraviolet excitation near 327 nm, they observed short-lived fluorescence primarily in blue-cone droplets. Under blue excitation at 460–490 nm, both green-cone droplets and P-type double-cone droplets fluoresced. Red-cone droplets fluoresced under green excitation at 520–550 nm.

The apparent difference between these observations may reflect differences in excitation wavelength, emission filters, exposure time, or species-specific droplet composition. Ohtsuka [37] used near-ultraviolet excitation extending toward 380 nm and detected longer-wavelength emission above approximately 460 nm. Kram et al. [3] used narrower channels optimized for classifying cone types rather than measuring complete excitation–emission spectra. Their ultraviolet channel may therefore not have detected the longer-wavelength emission described by Ohtsuka [37], particularly if excitation of the relevant droplets was more efficient near 365–380 nm than at 327 nm.

Together, these observations establish that several avian oil-droplet classes contain short-wavelength-excitable fluorescent material and that chicken P-type double-cone droplets fluoresce under blue excitation. They do not yet demonstrate that ultraviolet-induced emission from a P-type droplet is captured by the LWS pigment or produces a measurable double-cone response.

### 2.10. A Possible Fluorescence-Mediated Double-Cone Pathway

In a fluorescence-mediated pathway, near-ultraviolet radiation would first pass through the ocular media and reach the photoreceptor layer. A fluorophore within the P-type oil droplet would absorb part of this radiation and emit visible photons through a Stokes shift.

Because the droplet lies directly proximal to the outer segment, some of the emitted photons could enter the outer segment of the same double-cone principal member. If the fluorescence spectrum overlaps the absorption spectrum of the LWS pigment, these photons could initiate conventional phototransduction.

The resulting signal would probably not be encoded as a separate ultraviolet color. The LWS pigment would respond to a visible photon regardless of whether that photon originated in the environment or was generated locally by fluorescence. Ultraviolet radiation could therefore contribute to an achromatic double-cone signal. An ultraviolet-reflecting object might appear brighter or generate increased luminance contrast.

The mechanism should be distinguished from direct β-band activation of the LWS pigment. During β-band activation, the UV photon is absorbed directly by the visual pigment. During fluorescence-mediated activation, the UV photon is absorbed by the droplet fluorophore and subsequently replaced by a visible emitted photon.

Fluorescence is emitted in multiple directions, and much of the absorbed energy may be lost nonradiatively. Only a fraction of the emitted light would reach the outer segment. The pathway is therefore unlikely to equal the sensitivity of a dedicated SWS1 cone. It could nevertheless influence the luminance signal under sufficiently high UV irradiance, especially because the fluorophore is concentrated near the outer segment (Figure 1, panel 5).

This pathway is most plausibly relevant under photopic or upper-mesopic conditions, when double cones are functional and ultraviolet photon flux is high. It is less likely to explain fully scotopic UV behavior, particularly in rod-dominated nocturnal birds.

### 2.11. Ultraviolet-Sensitive OPN5 in the Inner Retina

Classical rods and cones are not the only photosensitive cells in the vertebrate retina. Several nonvisual opsins are expressed in inner-retinal neurons and glial cells. Of particular relevance is opsin 5, also called neuropsin or OPN5.

Chicken OPN5 is a bistable ultraviolet-sensitive pigment that signals through Gi-family G proteins. Its UV-sensitive state has an absorption maximum near 360 nm, providing a molecular mechanism for direct ultraviolet photoreception outside conventional rods and cones [39].

OPN5 and OPN3 expression has been detected in developing chicken inner retinal cells and Müller glia. OPN5-like immunoreactivity appears in the retinal ganglion-cell region and in cells extending through the developing nuclear layers. Both OPN3 and OPN5 have also been detected in primary cultures of chicken Müller cells [40].

The presence of OPN5 demonstrates that ultraviolet radiation can be detected directly by non-rod, non-cone retinal cells. However, expression of a UV-sensitive opsin does not establish participation in image-forming vision. OPN5 has been associated more strongly with local retinal regulation, circadian photoentrainment, development, vascular regulation, and seasonal physiology than with spatially resolved visual perception [39,41,42].

OPN5-mediated responses could nevertheless modify conventional visual processing by altering neurotransmission, retinal adaptation, dopamine signaling, circadian state, or the excitability of inner-retinal circuits. Thus, ultraviolet radiation may influence retinal function via OPN5 without producing a conscious perception of ultraviolet light.

### 2.12. Melanopsin and Other Photosensitive Retinal Molecules

Melanopsin, encoded by OPN4, is expressed in intrinsically photosensitive retinal ganglion cells and, in nonmammalian vertebrates, in several additional retinal cell populations. These cells can respond to light without direct rod or cone input and contribute to circadian entrainment, pupillary control, and other light-dependent functions [43].

Melanopsin is principally sensitive to blue-cyan light rather than ultraviolet radiation. It should therefore not be considered a primary UV receptor. Nevertheless, melanopsin pathways may influence retinal adaptation, brightness assessment, and behavioral responses to broad-spectrum illumination [43]

Cryptochromes and other photosensitive molecules are also expressed in avian retinal cells. Their functions include circadian regulation and light-dependent modulation of cellular physiology. In some cases, these molecules may participate in magnetoreceptive processes [44]. However, their contribution to image-forming ultraviolet perception remains uncertain.

### 2.13. Photosensitive Müller Glia

Müller glial cells span almost the entire thickness of the retina and interact with photoreceptors, bipolar cells, amacrine cells, ganglion cells, and retinal blood or metabolic-support systems. They regulate extracellular ions, neurotransmitter recycling, water balance, metabolic support, and neuronal excitability.

Avian Müller glia express photosensitive opsins, including OPN3 and OPN5. Cultured chicken Müller cells exhibit intrinsic light-evoked calcium responses, particularly to blue illumination, consistent with functional nonvisual photopigment signaling [40,45].

Because OPN5 is UV-sensitive, Müller glia may also respond directly to ultraviolet radiation. Such responses could affect retinal adaptation, cellular metabolism, neurotransmitter clearance, circadian physiology, or the gain of neighboring neural circuits. Nevertheless, there is currently no evidence that Müller cells independently encode a spatial ultraviolet image or directly generate image-forming UV vision.

Nonvisual photoreception should therefore be distinguished from image-forming perception. A bird may exhibit a retinal biochemical or physiological response to ultraviolet light without necessarily perceiving an ultraviolet object, pattern, or color.

### 2.14. Multiple Pathways Across Illumination Conditions

The available evidence suggests that ultraviolet radiation may influence the avian retina through several mechanisms operating across different illumination ranges.

Under bright photopic conditions, the SWS1 cone is the principal receptor for chromatic UV or violet vision. At the same time, high UV photon flux may activate the β-bands of other cone pigments or generate visible fluorescence in double-cone oil droplets. These additional routes would be expected to contribute primarily to luminance or temporal processing rather than to a distinct UV color channel.

Under mesopic conditions, rods and cones may operate simultaneously. The balance among SWS1 input, other cone β-bands, double-cone fluorescence, and rhodopsin β-band activation will depend on the intensity and spectral composition of the illumination, as well as retinal adaptation and, where present, retinomotor positioning.

Under fully scotopic conditions, the contributions of conventional cones should be greatly reduced. Rod rhodopsin becomes the most plausible receptor for image-forming UV detection, especially in highly rod-dominated nocturnal species such as owls. Inner-retinal opsins and other photosensitive molecules may continue to influence retinal physiology, but their contribution to spatial vision is uncertain.

OPN5 may respond directly to ultraviolet radiation, whereas OPN3, melanopsin, and other inner-retinal photosensitive molecules may contribute to responses elicited by the broader spectral composition of illumination. Their principal outputs are more likely to involve local modulation and non-image-forming functions than conventional visual perception.

### 2.15. Candidate Species for Comparative Investigation

Chickens provide the strongest initial model for testing double-cone fluorescence. Ohtsuka [37] reported UV-induced fluorescence in chicken retinal oil droplets, Kram et al. [3] independently demonstrated double-cone droplet fluorescence under blue excitation, and Rubene et al. [26] showed that UV illumination affects photopic temporal resolution.

Pied flycatchers and other passerines contain abundant double cones with pale P-type droplets. Their retinal organization provides a suitable anatomical substrate for fluorescence-mediated UV luminance sensitivity. However, many passerines also possess functional UVS SWS1 cones, requiring receptor-specific separation of the canonical and noncanonical responses.

Gentoo and Little penguins possess UV-transmitting ocular media but lack a known UV-maximized SWS1 pigment. They may therefore provide useful models for testing cone β-bands and double-cone fluorescence. King penguins (*Aptenodytes patagonicus*) are less suitable because their lenses strongly attenuate wavelengths below approximately 370 nm [13].

Owls are valuable for investigating rod β-band sensitivity because the species examined lack functional SWS1 cones and possess rod-dominated retinas with UV-transmitting ocular media [29,30]. They are less suitable as primary evidence for double-cone fluorescence unless a separate photopic UV response can be demonstrated.

Turtles remain relevant because Ohtsuka [37,38] directly demonstrated UV-induced visible fluorescence in droplets associated with red-sensitive cones. Similar mechanisms may occur in other reptiles, amphibians, fish, monotremes, or marsupials possessing pale oil droplets in LWS cones or double cones [21].

## 3. Experimental Approaches

The relative contribution of these pathways cannot be determined from behavioral UV sensitivity alone. Future experiments should combine behavioral testing with receptor-specific physiology.

Behavioral tests should be performed separately under photopic, mesopic, and scotopic conditions. Rapid flicker detection under bright light would favor cone or double-cone pathways, whereas a slow response enhanced by dark adaptation would favor rods.

Complete excitation–emission matrices should be obtained from isolated P-type droplets. The emission spectrum should then be compared with the absorption spectrum of the double-cone LWS pigment. Measurements at a single excitation wavelength are insufficient because they may miss the relevant excitation maximum.

The most direct electrophysiological approach would be single-cell recording from morphologically identified double-cone principal members in an ex vivo retinal preparation during narrow-band UV stimulation. Intracellular, patch-clamp, or suction-electrode recordings would be more informative than whole-retina electroretinography because they could assign the response to a specific receptor class. Electroretinography could nevertheless provide an initial action spectrum for screening candidate wavelengths. SWS1-cone input should be reduced by chromatic adaptation or isolated pharmacologically, and rod activity should be suppressed by photopic background illumination.

The fluorescence mechanism could be tested by photobleaching, quenching, removal, or optical bypassing of the P-type droplet. A selective reduction in the UV-evoked response, while preserving direct visible-light sensitivity, would support fluorescence-mediated activation.

Action-spectrum measurements are essential. A response matching the β-band of a visual pigment would support direct pigment absorption. A response matching the excitation spectrum of the droplet fluorophore would support fluorescence. A response near the OPN5 maximum, especially in isolated inner-retinal or glial cells, would support OPN5-mediated photoreception.

Finally, physiological detection should be separated from visual perception. Demonstration of light-induced calcium changes in Müller glia or inner-retinal neurons establishes photosensitivity but not necessarily image-forming vision. Behavioral discrimination of spatial patterns, luminance, or color is required to demonstrate perceptual involvement.

## 4. Conclusions

Avian ultraviolet sensitivity is not necessarily a unitary function mediated exclusively by SWS1 cones. Instead, ultraviolet radiation may enter the avian retina through several pathways that differ in receptor identity, illumination range, temporal properties, and functional output.

Under photopic conditions, SWS1 cones provide the principal basis for chromatic UV or violet vision. The β-bands of other cone pigments may contribute at high irradiance, while wavelength-shifting fluorescence in double-cone oil droplets may provide an additional route into the achromatic luminance system. Behavioral evidence that ultraviolet illumination increases chicken temporal resolution is consistent with UV input to a rapid photopic pathway, although the receptor has not been isolated [26].

Under scotopic conditions, especially in owls lacking functional SWS1 cones, the β-band of rhodopsin provides a more plausible pathway. The strong predominance of rods in owl retinas supports this interpretation [29,30]. Retinomotor movements may further shift the balance between rod and cone pathways in some species, although ultrastructural studies found little evidence of pronounced photomechanical movements in the great horned or barred owl [30,36].

In parallel, ultraviolet-sensitive OPN5 in inner-retinal neurons and Müller glia provides a molecular pathway for noncanonical retinal UV photoreception [39,40]. Such responses may regulate retinal physiology without necessarily participating directly in image-forming vision.

Fluorescence in double-cone P-type droplets may be considered one candidate within a broader group of noncanonical UV-detection mechanisms. Ohtsuka reported UV-induced visible fluorescence in chicken retinal oil droplets [37], whereas Kram et al. demonstrated fluorescence of chicken P-type double-cone droplets under blue excitation [3]. These observations provide an optical basis for investigating the proposed mechanism but do not demonstrate ultraviolet-induced activation of double cones. Whether fluorescence from a P-type droplet is sufficiently intense to activate the adjacent LWS outer segment remains to be determined.

These mechanisms are not mutually exclusive. Their relative contributions may depend on irradiance, adaptation state, ocular-media transmission, receptor abundance, retinal position, and behavioral task. Future investigations should combine controlled photopic, mesopic, and scotopic stimulation with action-spectrum measurements, receptor-specific electrophysiology, fluorescence spectroscopy, and behavioral discrimination rather than infer the responsible pathway from UV sensitivity alone.

## Data Availability

No new data were created or analyzed in this study. Data sharing is not applicable to this article.

## Abbreviations

The following abbreviations are used in this manuscript:

Gi: inhibitory guanine nucleotide-binding protein
LWS: long-wavelength-sensitive
OPN3: opsin 3 (encephalopsin or panopsin)
OPN4: opsin 4 (melanopsin)
OPN5: opsin 5 (neuropsin)
RH2: rhodopsin-like 2, or middle-wavelength-sensitive cone opsin
SWS1: short-wavelength-sensitive type 1
SWS2: short-wavelength-sensitive type 2
UV: ultraviolet
UVS: ultraviolet-sensitive
VS: violet-sensitive
